# Mr. Ring, on Cow-Pox

**Published:** 1801-03

**Authors:** John Ring

**Affiliations:** New Street, Hanover Square


### *To the Editors of the Medical and Phyfical Journal.*

Gentlemen,

YOUR impartiality, in admitting into your very interefting Journal, whatever tends to prove or difprove the utility of Vaccine Inoculation, deferves univerfal applaufe.

In your laft Number appeared a communication from Dr. Woodforde, not of the moft favourable kind to this queftion. But, whatever effect it may produce, I am too well acquainted with the author, and with the rectitude of his intentions, to doubt that his motives in publifhing the cafe were honourable.

In this inftance, a perfon who was fuppofed to have had the Cow-pox twenty-eight years ago, lately had the Small-pox; which, Dr. W. obferves, feems to militate againft the permanent preventive influence of the Cow-pox.

He fays, the above hiftory may be confidered as an addition to, and illuftration of, the two cafes communicated by Dr. John Sims in your Journal. It is therefore but juftice to the new practice to remark, that Dr. S. has, fince that time, candidly acknowledged, in the fame Journal, a conviction of his error, confeffing, he believed the matter ufed in vaccine inoculation, a real preventive of the fmall-pox. He has alfo figned the teftimonial, which declares, that in the opinion of the phyficians and furgeons, whofe names are under-figned, thofe who have had the Cow-pox are perfectly fecure from the future infection of the Small-pox.

The cafe of Mr Jacobs, publifhed by Dr. J. Sims, it is now well underftood, bears no refemblance to the genuine Cow-pox. It is the fpurious difeafe; againft which Dr. Jenner cautions every perfon to be on his guard, left it fhould create an idea of fecurity, which might prove delufive. But admitting, for the fake of argument, that the difeafe which is related by Dr. W. fprung fprung from matter generated in a puftule, or a fore, which had originally been of the true vaccine kind; what proof have we that this matter, when it was applied to the hands of the patient, and when it produced puftules, had not undergone a change? What proof have we, that, when this matter infected the patient in queftion, it had not degenerated, and loft all its original properties and fpecific virtue?

Of fuch a fpecies of degeneracy in the *Small-pox*, we meet with numerous inftances in the works of Dr. Jenner; inftances which occurred to very refpectable practitioners, who are now living to fubftantiate the facts.

If, then, the permanent preventive influence of the Cow-pox can bear as fevere a fcrutiny as that of the fmall-pox, which I am well perfuaded it can; this is all that the ingenious author of the paper under confideration, or any other reafonable and enlightened man, will expect; and if thefe obfervations tend in any degree to diffipate the doubts which Dr. W's cafe may have excited, I am perfectly convinced no one will read them with more pleafure than himfelf.

I hope this cafe will induce all medical men to inoculate with matter taken at an early period, and to refrain from the practice of taking it on the fourteenth and fifteenth day. I hope alfo, it will induce thofe perfons who imagine they have had the cafual Cow-pox, to confent to be inoculated for that difeafe, in order to preferve them from one infinitely more dreadful; and that my friend, Dr. Woodforde, will prevail on the fifter of his patient, to fubmit once more to this powerful prophylactic. Should it only prove a fecurity from the Small-pox for eight-and-twenty years more, it is worth while to try it at this time; fince it is probable, that before the expiration of half that period, the Small-pox will be known in this kingdom only by name.

It is by no means evident, that Dr. W's patient had formerly efcaped variolous infection, in confequence of having had the fuppofed vaccine difeafe; fince the fame thing often occurs in thofe who have never milked a cow, nor been fufpected of catching the difeafe in any other way. Dr. Bancroft, junior, lately informed me, that his brother was inoculated for the Small-pox eleven times, and once taken to a patient labouring under the difeafe, and inoculated in both arms with a large quantity of recent matter; but to no purpofe: Yet, he afterwards caught the Small-pox in the natural way.

Leaving every one to form his own opinion concerning permanent preventives, I beg leave to conclude with expreffing my opinion, that if medical men perform their duty, and fincerely unite in their endeavours to exterminate the difeafe, we fhall not long have occafion for a permanent preventive, or even for any any preventive at all; provided care is taken to enforce a per quarantine, when any veſſel arrives from foreign parts, where that worſt of plagues, the Small-pox, may happen to prevail. I am, &c.

*New Street, Hanover Square,*

*Feb.* 12, 1801.

JOHN RING.

